# Preferences and Barriers to Counseling for and Treatment of Intimate Partner Violence, Depression, Anxiety, and Posttraumatic Stress Disorder Among Postpartum Women: Study Protocol of the Cross-Sectional Study INVITE

**DOI:** 10.3389/fpsyt.2022.836350

**Published:** 2022-03-29

**Authors:** Lara Seefeld, Amera Mojahed, Freya Thiel, Julia Schellong, Susan Garthus-Niegel

**Affiliations:** ^1^Department of Psychotherapy and Psychosomatic Medicine, Faculty of Medicine, Technische Universität Dresden, Dresden, Germany; ^2^Institute and Policlinic of Occupational and Social Medicine, Faculty of Medicine, Technische Universität Dresden, Dresden, Germany; ^3^Institute for Systems Medicine (ISM), Faculty of Medicine, Medical School Hamburg, Hamburg, Germany; ^4^Department of Child Health and Development, Norwegian Institute of Public Health, Oslo, Norway

**Keywords:** intimate partner violence, depression, anxiety, PTSD, childbirth-related PTSD, postpartum women, INVITE study, study protocol

## Abstract

The cross-sectional study **INVITE** (**IN**timate partner **VI**olence care and **T**reatment pr**E**ferences in postpartum women) aims to examine treatment and counseling preferences and barriers in relation to the experience of intimate partner violence (IPV), depression and anxiety, and (childbirth-related) posttraumatic stress disorder (PTSD) among postpartum women in Dresden, Germany. Currently, the INVITE study consists of an interim sample of *N* = 1,787 participants with *n* = 891 completed interviews. Recruitment is ongoing, targeting a community sample of at least *N* = 4,000 women who complete various quantitative questionnaires *via* telephone interviews at 3–4 months postpartum. The differences in rates of IPV, postpartum depression and anxiety, and/or (childbirth-related) PTSD as well as treatment and counseling preferences and barriers between affected and non-affected women will be assessed. Further, predisposing variables, past and present stress exposure, enabling resources, as well as past and present health will be examined as predictors of service preferences and barriers. In this study protocol, the theoretical background, methods, as well as preliminary results regarding sociodemographic characteristics and birth-related factors of the interim sample are presented and discussed in terms of their socio-political relevance. Simultaneously assessing IPV, postpartum depression and anxiety, and (childbirth-related) PTSD will facilitate exploring comorbidities and concomitant special needs of affected women. Results of the INVITE study will therefore set the ground for well-aimed development and improvement of treatment and counseling services for the respective target groups by informing health care professionals and policy makers about specific preferences and barriers to treatment. This will yield the possibility to tailor services to the needs of postpartum women.

## Introduction

Intimate partner violence (IPV), depressive and anxiety disorders, as well as posttraumatic stress disorder (PTSD) are three global health care burdens affecting women of reproductive age, with detrimental consequences for both the individual and society (1–5). Women have been shown to be especially vulnerable to the experience of IPV, depression and anxiety, and PTSD during pregnancy and the postpartum period (6). Although equal access to health care is a central goal in all European health-care systems, international studies show that this aim has not been accomplished yet (7, 8). Rather, previous studies revealed inter-individual differences in access to health care and actual health care utilization due to socioeconomic, cultural, and ethnic characteristics (9–11).

### Intimate Partner Violence

The first global health care burden, IPV against women, refers to any behavior within a present or former intimate relationship that causes physical, psychological, or sexual harm. These behaviors include any acts of physical violence (e.g., slapping, kicking, and beating), sexual violence (e.g., forced sexual intercourse and other forms of sexual coercion), psychological abuse (e.g., insults, constant humiliation, intimidation, and threats), and any controlling behaviors (e.g., isolating a person from family and friends, monitoring their movements, and restricting access to financial resources, employment, education, or medical care) (12).

Violence by an intimate partner has lasting adverse effects on women and children’s health, wellbeing, and relationships (13, 14). In affected women, IPV is associated with compromised mental health and increased suicidality (8, 15, 16). Depression, anxiety, PTSD, panic disorders, and substance abuse disorders have been documented as the most common psychological consequences of IPV for mothers during the perinatal period (17, 18). It is estimated, that nearly one in three women worldwide experiences IPV during her lifetime (19, 20). In Germany, it is estimated that 22% of women experience at least one form of physical and/or sexual intimate partner violence in their lifetime, and 3% experienced it during the past year. Psychological abuse by a current or former partner was experienced by 50% of women in Germany (21). Regarding the perinatal period, on average, one-quarter of pregnant women is exposed globally (22) and the prevalence for IPV during the first year postpartum ranges from 2% in Sweden to 58% in Iran (23).

### Postpartum Depression and Anxiety

Regarding the second health care burden, women suffering from postpartum depression and anxiety represent a high-risk population for many types of morbidity. The worldwide pooled prevalence for postpartum depression is 17.7% (24), while the prevalence of postpartum anxiety disorders ranges from 15 to 20% (25, 26). Thus, especially in the vulnerable postpartum period, these women may struggle having their physical and psychological health needs met (27, 28). Postpartum depression and anxiety disorders furthermore increase the mother’s risk of poor physical health, lower self-esteem, higher perceived stress, lower social functioning, relationship difficulties, and poorer overall psychological health (29, 30). The most common cause of death for women in the first year after giving birth is suicide, which in turn is strongly related to symptoms of postpartum depression (31, 32).

Maternal postpartum depression and anxiety are also associated with negative effects on the child. Increased risk of becoming a victim of child abuse and poorer child health practices are observed in children of mothers suffering from postpartum depression (33–35). Further, children of mothers with postpartum depression and anxiety disorders are more likely to show difficult early temperament and impaired development (36–39). Partners of affected women also experience greater levels of stress, depression, and anxiety (4, 40). Therefore, identification and support of women affected by mental health issues during the postpartum period is not only important to prevent negative consequences for the whole family. It can also help to reduce the potential costs for society pertaining to increased health care costs (41).

### Childbirth-Related Posttraumatic Stress Disorder

Another health care burden affecting postpartum women is PTSD due to traumatic childbirth, which is referred to as childbirth-related PTSD. There are varying estimates of the prevalence of childbirth-related PTSD, with studies reporting rates from 3.1 to 4.0% in community samples to 15.7–18.5% in high-risk groups [e.g., women experiencing severe complications during pregnancy and labor or experiencing fetal or infant loss; (42, 43)]. Childbirth-related PTSD or higher levels of posttraumatic stress symptoms related to childbirth have been suggested to be associated with a decreased likelihood of having further children or delaying a subsequent pregnancy (44, 45). It has also been proposed that a new pregnancy has the potential to reactivate the posttraumatic symptoms (46). Although this research field is still to be explored, previous studies have demonstrated its great adverse potential for the mother, her children, the relationship with her baby and the relationship with her partner (47–50). Prior studies found a prospective impact of childbirth-related PTSD symptoms on children’s social-emotional development, not initiating breastfeeding, less favorable child sleep, and low couple relationship satisfaction (3, 51–53).

### Association Between Intimate Partner Violence, Postpartum Depression and Anxiety, and (Childbirth-Related) Posttraumatic Stress Disorder

Intimate partner violence, postpartum depression and anxiety, and (childbirth-related) PTSD seem to be linked to each other. Firstly, IPV victimization is associated with PTSD (54, 55). Is has been argued that violence inflicted by an intimate partner has the potential to be even more psychologically damaging than violence inflicted by a stranger because it cannot be seen as a random attack. Instead, the perpetrator is someone the victim used to trust, so that the violence has to be interpreted as the purposeful intent to harm (56). Experiencing abuse can affect the victim on a cognitive, affective, and behavioral level and may lead to a higher risk of revictimization (57). In turn, repeatedly experiencing victimization like IPV further increases the risk for PTSD (58).

Secondly, comorbidity between depression, anxiety, and PTSD is high (48, 59–61), especially in the weeks prior to delivery and 6 weeks postpartum (62). According to systematic reviews, this link could partly be due to the fact that women with postpartum depression and anxiety symptoms have a high prevalence and increased odds of having experienced childhood abuse and neglect (63–65) as well as partner violence during their lifetime and the past year (6, 23) and therefore also have a higher risk of suffering from PTSD. Additionally, previous studies have shown that current IPV significantly increased the likelihood of reporting traumatic childbirth experiences (66), which also increase the likelihood of postpartum depression and anxiety (67) and may result in childbirth-related PTSD. However, especially the link between postpartum depression and childbirth-related PTSD still needs further research. While pre-existing depression is a strong risk factor for developing childbirth-related PTSD, pre-existing PTSD increases the risk for postpartum depression (68–70), questioning the temporal relationship of both disorders.

### Service Utilization Among Women Affected by Intimate Partner Violence, Postpartum Depression and Anxiety, and/or (Childbirth-Related) Posttraumatic Stress Disorder

Women who experience postpartum depression, anxiety, or (childbirth-related) PTSD and who are suffering from having an abusive relationship with their partner might be the least likely to seek help. Only 33% of women who are physically or sexually abused by their partner contact the authorities (21). Furthermore, women experiencing IPV often do not seek help because they consider it as not needed or not useful, or they indicate that the violence or situation is not serious enough (71). Therefore, it is crucial to raise awareness regarding the seriousness of violence, whether it is physical, sexual, and/or psychological. Because investment in prevention and intervention services for survivors remains inadequate (72), it is crucial to improve possibilities of disclosure within the health care system (73). This is underlined by the fact that physicians’ motivation and readiness to address IPV contrast with uncertainty and lack of awareness (74).

Additionally, many women affected by postpartum depression, anxiety, or (childbirth-related) PTSD do not recognize the mental state they are in and rather attribute their symptoms to a normal part of motherhood such as fatigue, relationship difficulties after becoming parents, or personal weakness. Their partner, family, and friends may share this opinion and often even discourage women from seeking help (75, 76). Thus, high-quality evidence is now needed on how maternity and mental health services should address these issues in order to improve health outcomes for women and their families in the postpartum period. Although empirical evidence demonstrates that postpartum depression, anxiety, and (childbirth-related) PTSD are treatable (77–82), many women still do not receive any or only non-guideline based treatment. For instance, postpartum anxiety can be concealed by postpartum depression and therefore overlooked (83), also because women affected by postpartum anxiety seek professional help less often than women with postpartum depression (84). In the case of childbirth-related PTSD, women are also often misdiagnosed with postpartum depression (61, 85) and guideline-based treatment for childbirth-related PTSD is not offered to them as a result. Additionally, most postpartum women express confusion about treatment options and often opt out of treatment, partly out of concern over the impact of medication on breast milk or fear of being stigmatized as a “bad mother” (86–88). Knowing that women often are the gatekeepers for health in their families (89, 90), it is particularly important to create a service system that meets their needs.

### The Current Study

**FIGURE 1:**
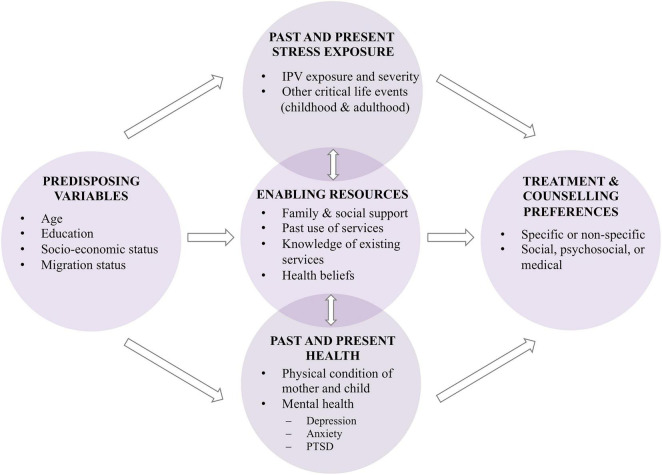
Theoretical framework of the INVITE study. Adapted from Andersen (91) and Liang et al. (92).

In order to investigate the associations between IPV, postpartum depression and anxiety, and (childbirth-related) PTSD and women’s individual treatment and counseling preferences and barriers, the current study applies concepts of Andersen’s Behavioral Model of Access to Health Care [BMCHC; (91)] and a model from Liang et al. (92) as the study’s theoretical framework (Figure 1). The BMCHC highlights the importance of predisposing variables (e.g., age, education, socio-economic status, and migration status), which can either be barriers or facilitators to service use. These predisposing variables may be associated with past and present stress exposure, available enabling resources, and past and present health, which in turn may also influence each other. Stress exposure might be due to IPV or other stressful life events during childhood or adulthood. In case of IPV, abuse severity can play an important role for the likelihood of help seeking (92, 93). Enabling resources include family and social network structures that may increase help-seeking behaviors, the subjective assessment of one’s own health needs, as well as prior use of services and interventions, which may have been effective. The past and present health status incorporates the mother’s mental health and the mother’s and child’s physical health. Within the theoretical model, these various factors culminate in women’s treatment and counseling preferences, which can be specific or non-specific to the issue and include service providers from the social, psychosocial, or medical sector.

The present cross-sectional study called **INVITE** (**IN**timate partner **VI**olence care and **T**reatment pr**E**ferences in postpartum women) intends to examine treatment and counseling preferences and barriers in relation to the experience of IPV, postpartum depression and anxiety, and (childbirth-related) PTSD among women delivering a child in Dresden, Germany at 3–4 months postpartum.

Based on the theoretical model, the main research questions are as follows:

1. Which factors predict higher/lower rates of IPV, depression and anxiety, and/or (childbirth-related) PTSD in postpartum women?
2. Are postpartum women who experience IPV, depression and anxiety, and/or (childbirth-related) PTSD less likely to seek treatment and counseling services compared to postpartum women who do not experience IPV, depression and anxiety, and/or (childbirth-related) PTSD?
3. Which type of treatment and counseling service, professional service provider, and service delivery mode do postpartum women who experience IPV, depression and anxiety, and/or (childbirth-related) PTSD prefer compared to postpartum women who do not experience IPV, depression and anxiety, and/or (childbirth-related) PTSD?
4. Does the severity of IPV, depression and anxiety, and/or (childbirth-related) PTSD in postpartum women predict the likelihood of service utilization?

Secondary research questions are as follows:

1. Do critical life events in the past predict the likelihood of service utilization in postpartum women?
2. Do family and social support predict the likelihood of service utilization in postpartum women?
3. Do knowledge about treatment and counseling services, the number of previous contacts with these services, and positive or negative experiences with them predict the likelihood of service utilization in postpartum women?
4. Do health beliefs about the usefulness of treatment and counseling services predict the likelihood of service utilization in postpartum women?
5. Do the mental or physical health of postpartum women or the physical health of their babies predict the likelihood of service utilization?

By filling the knowledge gap on how to improve postpartum women’s help seeking behavior, this study will improve health care services by informing health care professionals and policy makers about enabling factors as well as specific barriers to treatment. This in turn will yield the possibility to tailor services to the preferences of postpartum women.

## Methods

### Design and Procedure

The INVITE study is a cross-sectional study targeting a community sample of postpartum women in and around Dresden, Germany. Inclusion criteria are the birth of one or more children within the last 3--4 months and sufficient German or English skills to take part in the study. Women with a stillborn baby or an infant who died after birth are not excluded. Recruitment for the INVITE study started in November 2020. Women are either recruited at the maternity wards of two large hospitals in Dresden, at midwife (antenatal) appointments approximately 6 weeks prior to the planned delivery date, at birth information events of the hospitals, or at freestanding birth centers.^1^ Student assistants approach all mothers on the wards, at the antenatal clinics, and at the information events. At one hospital and the birth centers, women are approached by midwives instead. Recruitment therefore covers all maternity hospitals and most birth centers in Dresden, which offer different levels of specialization for high and low risk pregnancies, including pre-term deliveries. It ensures that almost all women delivering in Dresden are informed about the INVITE study and facilitates assembling a representative sample of postpartum women.

Recruitment procedures vary between the different hospitals and birth centers to accommodate their respective routines and preferences. Nevertheless, all women receive written information about the study’s aims and procedures as well as a small chocolate as an incentive. Women who agree to participate are asked to provide written informed consent. Unfortunately, the recruitment method had to be altered temporarily due to the COVID-19 pandemic. During lockdowns, all hospital visitation was prohibited and thus student assistants were not allowed to enter the hospitals for recruitment. Instead, nurses on the maternity wards and midwives at the antenatal appointments gave out the study material to interested women. This resulted in student assistants’ in-person recruitment starting December 2020 in one hospital, March 2021 in a second hospital, and July 2021 in the two remaining hospitals.

Ten weeks after the birth of their child or their expected delivery date, women are contacted to schedule an appointment for a standardized telephone interview, which lasts for approximately 1 h. The appointment takes place 3–4 months following birth, thus after completion of the puerperal period and at a time at which women are usually settling into everyday life with their newborn. An interview was chosen as our data collection method over an online or paper-pencil questionnaire for several reasons. Firstly, by calling and asking the women for the actual birth date of their child, we verify that all participants answer the questions 3–4 months after birth, even if they were recruited during pregnancy and we only know the expected delivery date. Secondly, by leading the women through the questions and being able to resolve any ambiguity straight away, the interview is less demanding for the participants than an online or paper-pencil questionnaire and therefore more suitable for postpartum mothers with a newborn that needs their attention, and for women whose mother tongue is not German or English. This assessment method also leads to higher data quality and less missing values, because questions can be explained to women who might not understand them correctly and hesitant women not wanting to answer questions due to privacy concerns can be assured of the pseudonymization strategy and motivated to answer all questions. Thirdly, a telephone interview is more personal than filling out a questionnaire, which makes it easier to transport the importance of the study to participating women and therefore reduces dropout.

After the interview, all women—regardless of whether or not they are affected by IPV, postpartum depression and anxiety, and/or (childbirth-related) PTSD—are offered a list with suitable treatment and counseling services in Dresden. Monetary compensation for study participation pertains to 20€. Women who were approached by student assistants and not the midwives received part of the compensation for participation (i.e., 5€ of 20€) at the time of recruitment.

### Materials

The INVITE study includes various quantitative questionnaires to assess the constructs of interest. Whenever possible, standardized and validated instruments with good psychometric properties were included and pertain to basic demographic and socioeconomic factors, pregnancy complications, birth-related medical information (e.g., mode of birth and birth complications), birth experience and childbirth-related posttraumatic stress symptoms, questions regarding the women’s and child’s physical health, critical life events, past and current mental health difficulties and treatments, symptoms of depression, anxiety, and non-birth-related PTSD, experience of IPV, social support, and resilience. Additionally, women are asked about their treatment and counseling preferences in case of experience of IPV, postpartum symptoms of depression, anxiety, or (childbirth-related) PTSD, barriers to service utilization, and their knowledge about suitable services. Women who are not affected by any of these symptoms or problems are asked to imagine the respective situation and answer the questions as if they were affected. Comparing affected to non-affected women will allow us to explore why affected women might not seek appropriate treatment.

**FIGURE 2:**
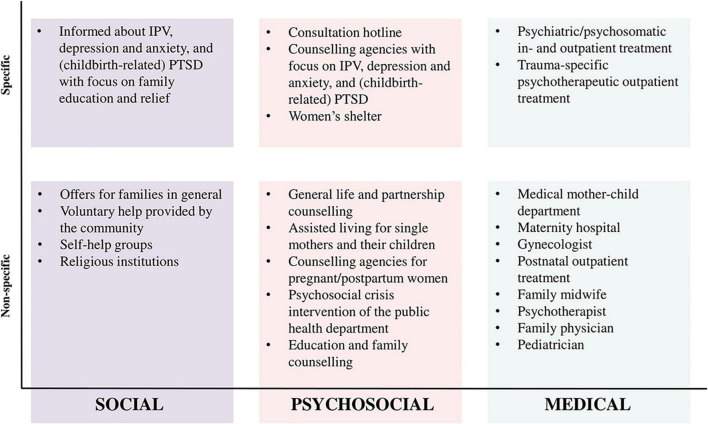
Treatment dimensions of the German support system.

Treatment and counseling services can range from very specific options tailored to IPV, postpartum depression and anxiety, and/or (childbirth-related) PTSD to more general services for all women such as the family physician. Possible approaches to treatment and counseling can be social, psychosocial, or medical. Figure 2 shows the different dimensions of treatment within the German support system. Regarding treatment and counseling preferences, women are asked to indicate how likely it is that they would pick a specific service if they are or imagine they were affected by IPV, postpartum depression and anxiety, and/or (childbirth-related) PTSD (“not at all,” “rather no,” “rather yes,” and “definitely”).

In the following, we present the instruments utilized to assess the main research questions. A comprehensive list of all assessed constructs can be found in Table 1. As the INVITE study collaborates with the International Survey of Childbirth-Related Trauma (94), a large, international project which examines birth trauma in different cultures around the world, some of our constructs were aligned with the mandatory measures of the INTERSECT study.

**TABLE 1:** Constructs and instruments in the INVITE study.

Constructs	Instruments
**Socio-demographic and socio-economic factors**	
Country of birth and mother tongue	Questions derived from the DREAM study (95) and the German National Cohort (96)
Nationality and residency status	Questions derived from the DREAM study (95), and the German National Cohort (96)
Place of residence (urban, sub-urban, or rural)	Question derived from the INTERSECT study (94)
Education and professional qualifications	Questions derived from the German National Cohort (96)
Marital status and current partnership	Questions derived from the DREAM study (95), the Socio-Economic Panel (97), and self-generated questions
Income	Questions derived from (98)
**Somatic factors**	
Current somatic health	Question derived from the DREAM study (95) and the ABC study [e.g., (52, 99)]
**Former pregnancies**	
Number of other children and corresponding date(s) of birth	Questions derived from the INTERSECT study (94)
Traumatic birth of other children	Questions derived from the INTERSECT study (94)
**Recent pregnancy and birth**	
Complications during pregnancy	Maternity records [“Mutterpass^4^”; (100)]
Singleton or multiple pregnancy	Self-generated question
Gestational week at time of birth	Maternity records [“Mutterpass”; (100)]
Sex of child	Self-generated question
Desirability of pregnancy and whether it was planned	Self-generated question
Mode of birth	Questions derived from the INTERSECT study (94)
Medical complications during birth (mother)	Questions derived from the INTERSECT study (94)
Medical complications during birth (child)	Questions derived from the INTERSECT study (94)
Support during birth	Questions derived from the INTERSECT study (94)
Birth satisfaction	Birth Satisfaction Scale-Revised [BSS-R; (101)]
Traumatic prior or recent birth	Questions derived from the INTERSECT study (94)
Childbirth-related Posttraumatic Stress Disorder	City Birth Trauma Scale (102, 103)
**Child-related factors**	
Breastfeeding	Self-generated questions
Child health and development	Questions derived from the DREAM study (95) and the child’s medical records [“Kinderuntersuchungsheft”; (104)]
**Mental factors**	
Former and current mental disorders and treatments	Questions derived from the INTERSECT study (94) and self-generated questions
Critical life events	Life Events Questionnaire (105)
Symptoms of Depression	Edinburgh Postnatal Depression Scale [EPDS; (106, 107)]
Symptoms of Anxiety	Subscale “anxiety” of the Symptom Checklist Revised [SCL-90-R; (108)]
Symptoms of non-birth-related Posttraumatic Stress Disorder	Primary Care PTSD Screen for DSM-V (109)
Traumatic events	Trauma list of the Posttraumatic Diagnostic Scale for DSM-5 (110)
Resilience	Resilienzskala [RS-11; (111)]
**IPV**	
Attitudes toward violence against women	Questions derived from the Special Eurobarometer 449: Report for Gender-Based Violence (112)
Experience of controlling behaviors, psychological, physical, and sexual violence, and coping and impact measures	The Violence Against Women Instrument [VAWI; (113)]
**Treatment/intervention and counseling preferences**	
Preferred treatment and counseling services	Self-generated questions based on Simhi et al. (114)
Knowledge of services	Self-generated questions
Preferred service delivery mode	Self-generated questions
Barriers to seeking treatment and counseling services	Self-generated questions on the basis of the Health Beliefs Model (115–122)
**Social factors**	
Social support	Short version of the Social Support Questionnaire [“Fragebogen zur Sozialen Unterstützung” F-SozU-14; (123)]
**COVID-19-related factors**	
Burden due to COVID-19	Self-generated question

*^4^Booklet which remains with the mother throughout pregnancy and after birth. Midwives and doctors document results from prenatal appointments and birth therein.*

#### Violence Against Women Instrument

The Violence against Women Instrument [VAWI; (124)] is used to assess any experiences of IPV. All questions pertain to violence perpetrated by a current or any former partner. The instrument consists of behavior-specific items related to psychological (four items), physical (six items), and sexual IPV (three items). The physical violence items are further divided into “moderate” (the two first items) and “severe” (the following four items) violence based on the likelihood of physical injury. Respondents are categorized as having been exposed to IPV if they answer any question affirmatively (“yes” coded as 1, or “no” coded as 0), with separate indicators created for each type of IPV (113). For each question, respondents are asked whether they had experienced the specific act during the *past year* and/or *earlier in life*. The frequency of experienced IPV is also assessed and coded as 1 (“once”), 2 (“a few times”), and 3 (“many times”). Cronbach’s α coefficient was found to be satisfactory for the subscales in the VAWI conceptual model: 0.79 (psychological scale), 0.80 (physical scale), 0.72 (sexual scale), and 0.88 (total scale) (125). The instrument was translated to German according to the suggestions of the World Health Organization (126) and first checked by researchers involved in the present study who could compare the English version with its translation (expert panel). These researchers then conducted lengthy oral back translation with step-by-step discussion of each question with non-experts fluent in German and English to decide on a final version of the instrument. The research team is planning to conduct a validation study for the translated German version of the instrument.

#### Edinburgh Postnatal Depression Scale

To assess postpartum depression, we use the Edinburgh Postnatal Depression Scale [EPDS; (106, 107)]. The EPDS was developed as a screening tool for postnatal depression, measuring depressive symptoms during the last week. The scale consists of 10 items rated on a 4-point scale, ranging from 0 to 3, with a maximum score of 30 where higher scores indicate more severe symptoms of depression (127). Cronbach’s α for the German version is high [α = 0.81; (106)].

#### Symptom Checklist-Revised

To measure postpartum anxiety, we use the “anxiety” subscale of the Symptom Checklist-Revised [SCL-90-R; (108)]. It consists of 10 items pertaining to symptoms during the last 7 days. Each item is rated on a 5-point Likert scale, ranging from 0 (“not at all”) to 4 (“extremely”). The total score ranges from 0 to 40 with higher scores indicating more severe levels of anxiety. Cronbach’s α for the subscale “anxiety” is high [α = 0.84; (108)].

#### City Birth Trauma Scale

The City Birth Trauma Scale [City BiTS; (102, 103)] is used to assess childbirth-related PTSD symptoms and consists of 22 questions, which map onto DSM-5 diagnostic criteria and therefore measure re-experiencing or avoidance of the traumatic event, negative cognitions and mood, and hyperarousal. Symptoms are rated according to frequency over the last week and scored on a scale ranging from 0 (“not at all”) to 3 (“5 or more times”). A higher score indicates more and/or higher frequency of symptoms of childbirth-related PTSD. Diagnostic criterion A items (i.e., perceived threat of serious injuries to mother or child or perceived threat that the mother or child might die) are scored on a yes/no scale. Distress, disability, and potential physical causes are rated as yes/no/maybe. Cronbach’s α for the total German scale is high [α = 0.92; (103)]. In addition to the City BiTS, women were asked to indicate how traumatic they found their recent and potential earlier births on a scale from 0 (not at all traumatic) to 10 (very traumatic).

#### Primary Care Posttraumatic Stress Disorder Screen for DSM-5

A short version of the Primary Care PTSD Screen for DSM-5 [PC-PTSD-5; (109, 128)] is used to assess PTSD symptoms due to non-birth-related events. The measure consists of five items and begins with a question designed to assess whether the respondent has had any exposure to traumatic events. If a respondent denies exposure, the PC-PTSD-5 is complete with a score of 0. If a respondent indicates that they have experienced a traumatic event over the course of their life, the respondent is instructed to respond to five additional yes/no questions about how that trauma exposure has affected them over the past month. However, in the INVITE study, the PC-PTSD-5 was adjusted so that women are asked to think of an event that was “so frightening, horrible, or upsetting” that they may have experienced any of the five symptoms during the past month. This way, all women are asked for possible PTSD symptoms. This adjustment was done according to Harrison et al. (129). Additionally, if a woman answers “yes” to any of the items, she is asked how stressful she found this symptom.

All data are collected and managed using Research Electronic Data Capture (REDCap), which is a secure, web-based application for data capture within research studies, hosted at the “Koordinierungszentrum für Klinische Studien” at the Faculty of Medicine of the Technische Universität Dresden, Germany (130, 131).

### Sample

**FIGURE 3:**
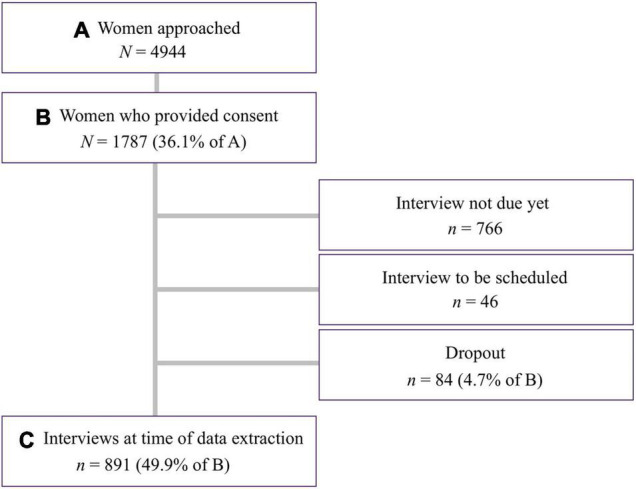
Flow chart of the INVITE study. Data extraction took place on 29 October 2021.

A flow chart of the INVITE study is depicted in Figure 3. By 29 October 2021, *N* = 4,944 women were approached and informed about the study. In total, *N* = 1,787 of the approached women gave written informed consent and were included in the interim sample (recruitment ongoing). This corresponds to a participation rate of 36.1%. Some of these women (*n* = 766) were not due to be interviewed yet at the time of data extraction because they gave birth less than 3 months ago and others still needed to be contacted to arrange an interview (*n* = 46). Only *n* = 84 (4.7%) of the women who gave consent dropped out of the study before the interview took place, because they could not be reached or indicated not having time to complete the interview. In total, *N* = 891 interviews (49.9% of those who gave consent) were completed by the end of October 2021. To calculate the response rate, we compared the proportion of approached women who had their baby before 7 August 2021 (i.e., more than 3 months before data extraction; *N* = 3,502) with the proportion of women who participated in the interview. This resulted in a response rate of 25.4%.

The response rate will likely increase over the course of the study because our recruitment method had to be altered temporarily due to the COVID-19 pandemic. As described above, during lockdowns, student assistants were prohibited from entering the hospitals for recruitment and thus, maternity ward nurses and midwives at the antenatal appointments gave out the study material instead. There are several reasons why this procedure resulted in fewer participating women. For instance, nurses and midwives were not able to explain the study in detail, answer questions, or give out the initial incentive of 5€. This might have resulted in women taking the materials home but forgetting about them. This altered procedure does not correspond with our intended recruitment method of proactive outreach, with which an entire population is individually addressed, contacted, and motivated to participate in the study, and may explain why the response rate is lower than anticipated. Since the implementation of proactive outreach in all four hospitals in July 2021, participation rates have increased to 60–80%. When calculating the response rate for the time when student assistants were recruiting in person in two out of four hospitals (i.e., from March 2021), the rate increases to 35.4% (*n* = 2,084 women were approached of which *n* = 737 completed the interview).

Table 2 shows where the participating women were recruited. The majority (75.3%) were recruited on the maternity wards of the two largest hospitals with a delivery room in Dresden. These are Level 1 and Level 2 hospitals, which means that they are equipped to handle high-risk pregnancies and pre-term births and have a neonatal intensive care unit. Most of the remaining women were recruited at antenatal appointments of the midwife-led outpatient clinics (23%) of the other two hospitals (both are certified as baby-friendly by the World Health Organization and UNICEF). Additionally, some women were recruited by midwives at two freestanding birth centers or contacted the study team directly to participate (1.7%).

**TABLE 2:** Places of recruitment of the INVITE study.

	Mothers (*n* = 891) *n* (%)
Maternity ward	
Level 1 maternity hospital	419 (47.0)
Level 2 maternity hospital	252 (28.3)
Midwife-led outpatient clinics for pregnant women (*n* = 2)	205 (23.0)
Freestanding birth centers (*n* = 2)	14 (1.6)
Contacted study team herself	1 (0.1)

*Level 1 and Level 2 hospitals have neonatal intensive care units.*

### Planned Data Analysis

To answer main research questions 1 and 4 as well as all secondary research questions, we will compute simple and multiple linear regression analyses. This will allow us to identify factors predicting higher/lower rates of IPV, depression and anxiety, and/or (childbirth-related) PTSD in postpartum women and to investigate factors predicting likelihood of service utilization. To answer main research question 2 and to examine potential differences between treatment and counseling preferences of postpartum women affected vs. those not affected by IPV, depression and anxiety, and/or (childbirth-related) PTSD, we will utilize one-way ANOVA and *post-hoc* tests to determine specific differences. To answer main research question 3 and to specify the type of treatment and counseling service, professional service provider, and service delivery mode postpartum women affected vs. not affected by IPV, depression and anxiety, and/or (childbirth-related) PTSD prefer, we will utilize one-way MANOVA and MANCOVA and *post-hoc* tests to identify specific differences. For the ANOVA, MANOVA, and MANCOVA, participating women will be divided into groups depending on their scores on the VAWI, EPDS, SCL-90-R, City BiTS, and PC-PTSD-5. Group allocation will be performed using the respective questionnaire’s cut-off score. Women scoring below the cut-off will be considered as not affected (i.e., they imagined being affected by any of the issues to answer the questions on treatment and counseling preferences).

### Power Analysis

An *a priori* power analysis was computed using G*Power 3.1.9.2 (132) to determine the required sample size. Because the main research questions investigate group differences between women with and without experience of IPV, postpartum depression and anxiety, and/or (childbirth-related) PTSD regarding treatment and counseling preferences, the power analysis was computed for independent samples *t*-tests. Other research questions will be investigated through multiple linear regression analyses—the interim sample is already large enough to detect small effects with sufficient power (results not shown). Results of the power analysis for independent samples *t*-tests indicated that a total sample of *n* = 1,360 women is needed to detect small effects (*d* = 0.2) with 80% (1-beta) power and alpha at 0.05, with a prevalence of 17.5% for postpartum depression and anxiety (24–26). Moreover, according to a recent meta-analysis, the prevalence of significant levels of childbirth-related posttraumatic stress symptoms is estimated at 13% (133), thus requiring a sample size of *n* = 1,740 to detect a small-sized effect with 80% power. For IPV, the prevalence for physical and sexual violence during the last 12 months is estimated at 3% in Germany (21), which results in an estimated required sample size of *n* = 6,678 to detect small-sized effects with a power of 80%. As it might not be feasible to recruit this many women for the study, we may only be able to detect medium (*d* = 0.5) or large effects (*d* = 0.8) for physical and sexual IPV, requiring samples of *n* = 1,072 and *n* = 424 women, respectively. For psychological violence by a current or former partner on the other hand, the prevalence is estimated at 50% in Germany (21), which corresponds to a required sample size of *n* = 788 to detect small effects.

Given these analyses, a minimum of *n* = 1,740 women has to be interviewed for our study to detect small effects for postpartum depression, anxiety, childbirth-related posttraumatic stress symptoms, and psychological IPV and medium effects for physical and sexual IPV with a power of at least 80% and alpha at 0.05. However, according to our current estimation, we will recruit at least *n* = 4,000 women until the end of the study’s funding period, which provides us with a highly powered study to detect even smaller effects and include potential confounding variables in our analyses as well.

Although recruitment is ongoing, we already have a large enough interim sample to compute multiple linear regression analyses examining risk and protective factors for IPV, postpartum depression and anxiety, and (childbirth-related) PTSD and examining the predictive value of several factors for service utilization.

## First Results

Below, preliminary results regarding the sample of the INVITE study are presented.

### Sociodemographic Characteristics

Preliminary sociodemographic characteristics of the sample are presented in Table 3. The mean age of participating mothers was 32.5 years (*SD* = 4.5; *Range* = 19--51). The majority (91.0%) was born in Germany and had German nationality (93.7%). Compared to Dresden’s general population,^2^ our sample had a slightly lower rate of women without German nationality [6.3 vs. 8.6%; (134)]. Among mothers without German nationality, most had a permanent residence status (69.6%). Nearly all participants were currently in a partnership (97.8%). The majority (64.6%) had a subject-related or higher education entrance qualification (A-level) and a university degree (48.1%). Compared to the general German (135) and Dresden population (136), our sample is characterized by a high educational and professional status. Slightly more than half the women were primiparous (51.2%) and on average, the interviews were held 12.1 weeks after birth (*SD* = 2.6; *Range* = 4–45).

**TABLE 3:** Sociodemographic characteristics of the INVITE study sample.

	Mothers (*n* = 891)
Age	32.5 ± 4.5 (19–51)
**Country of birth**	
Germany	811 (91.0)
Other	80 (9.0)
**Nationality**	
German	823 (92.4)
German and other	12 (1.3)
Other	56 (6.3)
**Residence status of mothers without German nationality**	
Permanent	39 (69.6)
Temporary (visa, residence permit, refugee status)	15 (26.8)
Seeking asylum	2 (3.6)
**Current partnership**	
Yes	871 (97.8)
No	20 (2.2)
**Education**	
No degree (yet)	4 (0.4)
Lower secondary education level 2	21 (2.4)
Secondary school certificate	218 (24.5)
Advanced technical college entrance qualification	71 (8.0)
Subject-related or higher education entrance qualification (A-level)	576 (64.6)
Missing data	1 (0.1)
**Professional qualification**	
No qualification (yet)	42 (4.7)
Occupational apprenticeship	325 (36.5)
Master of crafts	48 (5.4)
University degree	429 (48.1)
Doctoral degree	42 (4.7)
Missing data	5 (0.6)
**Net earnings/month of the whole household on average**	
Less than 1,250€	24 (2.7)
1,250–1,749€	51 (5.7)
1,750–2,249€	54 (6.1)
2,250–2,999€	128 (14.4)
3,000–3,999€	250 (28.1)
4,000–4,999€	197 (22.1)
More than 5,000€	183 (20.5)
Missing data	4 (0.4)
**Number of children**	
1	456 (51.2)
2	346 (38.8)
3	68 (7.6)
4	16 (1.8)
5 or more	4 (0.4)
Missing data	1 (0.1)
Time since birth at the time of the interview (in weeks)	12.1 ± 2.6 (4–45)

*n (%) or M ± SD (range).*

### Birth-Related Characteristics

Table 4 displays birth-related characteristics of the sample. Most mothers gave birth to one child (97.9%) with the rest giving birth to twins. The sex ratio of the children was nearly balanced with 50.2% being female. On average, women gave birth at 39.9 weeks of gestation (*SD* = 1.5; *Range* = 30--43) and the pre-term birth rate was 6.1%. The latter is slightly lower than the German average of 8.2%^3^ (137). Most mothers delivered vaginally (74.2%) and the rate of cesarean sections was 19.2%, which is low compared to the German average of 30.9% (137) and slightly lower than the Dresden average of 21.3% (138).

**TABLE 4:** Birth-related characteristics of the INVITE study sample.

	Mothers (*n* = 891)
**Number of delivered children**	
One	872 (97.9)
Twins	19 (2.1)
**Sex of child (*n* = 910)**	
Female	457 (50.2)
Male	453 (49.8)
Week of gestation at birth	39.9 ± 1.5 (30–43)
**Mode of birth**	
Vaginal birth	661 (74.2)
Vaginal operative birth (with forceps or vacuum extraction)	59 (6.6)
Planned cesarean section	106 (11.9)
Emergency cesarean section	65 (7.3)

*n (%) or M ± SD (range).*

## Discussion

Examining experiences of IPV, symptoms of depression and anxiety, and (childbirth-related) PTSD in postpartum women, the INVITE study is one of the first studies to assess and compare counseling and treatment preferences as well as barriers to help seeking among mothers affected by IPV and postpartum mental health problems vs. those who are not affected.

In sum, the INVITE study has various major strengths. It is one of the first studies to examine differential risk and protective factors for IPV, depression, anxiety, and (childbirth-related) PTSD in postpartum women, which will enable us to also explore comorbidities and concomitant special needs of affected women. Moreover, our study not only analyzes women’s knowledge on and previous experience with various German service providers, but also their preferences for these services and service delivery modes. Asking about different providers from the social, psychosocial, and medical sector will identify specific services that postpartum women may find especially helpful and should therefore be advertised and recommended to mothers experiencing IPV or showing signs of postpartum depression, anxiety, and/or (childbirth-related) PTSD. Furthermore, the INVITE study considers possible barriers to treatment and counseling services based on the Health Belief Model (120). This will enable us to identify reasons for a potentially low utilization of services among postpartum women and ways to improve existing services. Because several instruments (e.g., birth satisfaction and IPV) were not available in German and thus had to be translated by our team, we plan to conduct several validation studies. This way, we will further contribute to adequately assessing IPV and mental health related issues in postpartum women in German-speaking regions.

Additionally, due to our successful recruitment method, we are able to reach most women giving birth in Dresden. In fact, almost 5,000 women were approached for our study in the first 12 months of recruitment compared to 5,747 live births in Dresden in 2020 (139). By increasing the proportion of proactive outreach through our student assistants (instead of passive recruitment through hospital staff), we were able to steadily increase our participation rate to 60–80%. This is not yet reflected in the presented interim sample because proactive outreach was only implemented in all hospitals in July 2021 due to COVID-19-related restrictions. Despite this delay in recruitment, we would already be able to address several of our research questions.

Another strength of the INVITE study is the ability to capture a genuine image of the experience of IPV, postpartum depression and anxiety, and (childbirth-related) PTSD and respective treatment and counseling services among women with a migrant background in Germany. Previous research has often been limited in generalizability by under-representing migrant populations, especially in the context of IPV and mental health (140–142). Because women with a migrant background may encounter specific challenges regarding treatment and counseling [e.g., language barriers, needing an interpreter, and corresponding costs as well as additional expenditure of time; (143)], it is especially important to assess these women’s preferences and barriers to better tailor services to their needs. Offering the interview in both German and English has resulted in a participation rate of women with a migrant background which is close to the rate in the general population of Dresden.

Finally, the INVITE study collaborates with the INTERSECT study (94), which assesses birth trauma and childbirth-related PTSD across low-, middle-, and high-income countries and therefore offers the opportunity for cross-cultural comparisons on various variables, including prevalence of childbirth-related PTSD, differences in symptom presentation, and etiology. In addition, most participating countries also assess birth satisfaction and postpartum depression, and some investigate IPV, which will facilitate comparing prevalence and characteristics of these issues as well. Next to these data, we will have access to information about the context of maternity care in the respective countries (e.g., private vs. public health care, service providers, service utilization rate, and obstetric intervention rates). This information will enable us to interpret the cross-cultural comparisons of various factors against the background of care provided to postpartum women within these countries. Thus, while the INVITE study is of outmost national importance due to its detailed assessment of postpartum women in Germany, the collaboration with the INTERSECT study expands this importance to an international level.

First results on the interim sample show that women participating in the INVITE study are characterized by a rather high educational and professional level compared to the general German population (135) and the population of Dresden (136). This is in line with previous research on large epidemiological studies (95, 144, 145) and indicates that our results might not be generalizable to less educated samples. However, our sample is comprised of almost equal amounts of primiparous and multiparous women. This is important for differential analyses regarding birth-related characteristics, because parity has been identified as a significant covariate in some studies, for example on birth experience (146, 147). The interim sample further mostly includes mothers who had a vaginal birth as can be expected for Dresden (138), where the rate of cesarean sections is low compared to the national average. Moreover, the rate of pre-term births in our sample was slightly lower than the national average (137). This may be explained by pre-term babies needing extensive and time-consuming care and mothers of these babies therefore not feeling capable of doing a long telephone interview but could also be a normal variation in numbers.

To summarize, the data generated by the present study will set the ground for a set of future publications that in turn will serve for a well-aimed development of treatment and counseling services for the respective target groups. These publications will primarily answer our main and secondary research questions. Further publications in collaboration with the INTERSECT study are also planned in the context of providing cross-cultural comparisons. Moreover, validation studies will be published for the utilized instruments for IPV and birth satisfaction. This will further enhance future research methodology in the present context. Hence, the INVITE study will improve current health care system services by informing health care professionals and policy makers about specific barriers to treatment and will thus yield the possibility to tailor services to the preferences of postpartum women. Beyond that, we will deliver important information on how to prevent IPV, postpartum depression and anxiety, and (childbirth-related) PTSD in the first place. Through the telephone interviews and by offering women a list with suitable treatment and counseling services in Dresden, we create awareness and encourage treatment or preventive steps before women get to the point where those experiences are seriously influencing their quality of life and interaction with their families. Finally, the results of the INVITE study are not only important on a scientific level, but also of socio-political relevance. Informing policy makers and politicians about the results of the study may help generate more political interest in these pressing issues for women and their families. In particular information on specific services, which women find helpful may drive decision makers to rearrange attention and funding to these services as investing in prevention and delivering effective help decreases long-term health-care costs (41).

## Data Availability Statement

The raw data supporting the conclusions of this article will be made available by the authors, without undue reservation.

## Ethics Statement

The studies involving human participants were reviewed and approved by the Ethics Committee of the Faculty of Medicine of the Technische Universität Dresden. The patients/participants provided their written informed consent to participate in this study.

## Author Contributions

SG-N and JS acquired funding and were responsible for conception and design of the INVITE study as well as the coordination and supervision of the (ongoing) data collection. LS, AM, and FT supported the conduction of the study and data collection. AM and LS prepared the data for statistical analysis and wrote the first draft of the manuscript. LS performed the statistical analysis. All authors contributed to manuscript revision, read, and approved the submitted version.

## Conflict of Interest

The authors declare that the research was conducted in the absence of any commercial or financial relationships that could be construed as a potential conflict of interest.

## Publisher’s Note

All claims expressed in this article are solely those of the authors and do not necessarily represent those of their affiliated organizations, or those of the publisher, the editors and the reviewers. Any product that may be evaluated in this article, or claim that may be made by its manufacturer, is not guaranteed or endorsed by the publisher.

## Footnotes

1 Led mostly by midwives, these birth centers care for women with low-risk pregnancies who plan to deliver at the birth center or at home.

2 Due to large regional differences in the number of people without German nationality, the German average of 12.7% (148) is not comparable to local rates.

3 No rates for Dresden available.
